# Analysis of Erect-Panicle Japonica Rice in Northern China: Yield, Quality Status, and Quality Improvement Directions

**DOI:** 10.3390/plants13070926

**Published:** 2024-03-22

**Authors:** Bingchun Yan, Xinmei Jiang, Zhengjin Xu, Wenfu Chen, Xiaoyi Cheng, Hai Xu

**Affiliations:** 1 Rice Research Institute, Shenyang Agricultural University, Shenyang 110866, China; 2021200069@stu.syau.edu.cn (B.Y.); xuzhengjin@126.com (Z.X.); wfchen5512@126.com (W.C.); 2 National Institute of Biochar, Shenyang Agricultural University, Shenyang 110866, China; 2021500025@test.syau.edu.cn

**Keywords:** japonica rice, nitrogen-based fertilizer, northeastern China, yield, brown rice food quality

## Abstract

China is the only country that extensively cultivates the indica and japonica rice varieties, with the largest japonica rice production area being in northeast China. A study of the relationship between the yield and quality of japonica rice and the effect of nitrogen fertilizer application on this relationship is important. In this paper, we aimed to assess the current yield and quality of japonica rice in northeast China. We selected erect-panicle varieties as the test materials. Field experiments were conducted using different nitrogen fertilizer levels for two consecutive years to analyze the rice varieties’ yield, quality, interrelationship, and nitrogen fertilizer response. The average yield following high- and low-nitrogen treatments exceeded 10,000.00 kg/hm^2^, with a maximum of 12,285.63 kg/hm^2^. The high-yield–high-nitrogen treatment group had more panicles, a higher seed-setting rate, and a higher 1000-grain weight than the other groups. The high-yield–low-nitrogen group had a higher number of panicles and seed-setting rate than the other groups. The low-yield–high-nitrogen group had a lower number of whole grains, grain length-to-width ratio, and taste value than the other groups. The low-yield–low-nitrogen group had fewer primary branches than the other groups; excluding the primary branch-setting rate and 1000-grain weight, the values of the other panicle traits of the group were significantly higher than those of the other groups. The high-nitrogen–high-flavor group had lower panicle and spikelet numbers and higher spikelet fertility rates than the other groups. The low-nitrogen–high-flavor group had higher spikelet fertility rates and 1000-grain weight than the other groups. Compared to the other groups, the low-nitrogen–high-flavor group had a higher head rice yield, and the high-nitrogen–high-flavor group had a lower chalkiness rate. The main goal of the breeding and cultivation of high-yield and high-quality erect-panicle japonica rice in northern China is to achieve “dual high, dual low, and one high and one low” conditions, signifying a high yield with high or low nitrogen levels, low protein and amylose contents, high head rice rates, and low chalkiness. This study provides a new technique for enhancing the taste of northern erect-panicle japonica rice to promote the sustainable, high-yield, and high-quality development of japonica rice in northern China.

## 1. Introduction

Semi-dwarf varieties, hybrid rice, and the erect-panicle type are three breakthroughs in rice breeding in China [1]. Since the introduction of the first northern erect-panicle japonica variety, Liao Japonica 5, in the 1980s, erect-panicle japonica varieties have become popular in the north, especially in the southern northeast rice region, and have been vital in improving local rice yields [2]. Rice is the staple food of >60% of the Chinese population, and high yields have long been the priority of rice breeders in China. However, with the increase in scientific research, advances in production technology, and improvement in living standards, the goal of rice breeding has changed from a high yield to a high yield and high quality, with more attention being directed to quality, especially taste quality [3]. With the improvement in rice varieties, the nitrogen in rice grains was gradually reduced, and the quality of starch was improved [4]. The nitrogen utilization efficiency of japonica rice is lower than that of indica rice, and the continuous excessive nitrogen fertilizer input in japonica rice production leads to resource and energy waste and causes ecological and environmental problems [5,6]. After several decades of genetic improvement, erect-panicle japonica rice had a more positive effect on yield, harvest index, and the size of sink [7]; the recently developed erect-panicle-type varieties have significantly improved in grain set and quality with a high yield potential, surpassing the generally curved-panicle-type varieties (loose- or sparse-panicle types) [2]. At present, there are few studies on this aspect. In this paper, we aimed to analyze the current status of the yield and quality of erect-panicle japonica varieties promoted in terms of production, their interrelationships, and their corresponding nitrogen fertilization as well as explore potential directions for the future enhancement in rice quality. The findings of this study could help to promote the harmonization of the yield and quality of erect-panicle japonica rice in north China at a high level.

## 2. Results

### 2.1. Rice Production and Its Constituent Factors

**Figure 1:**
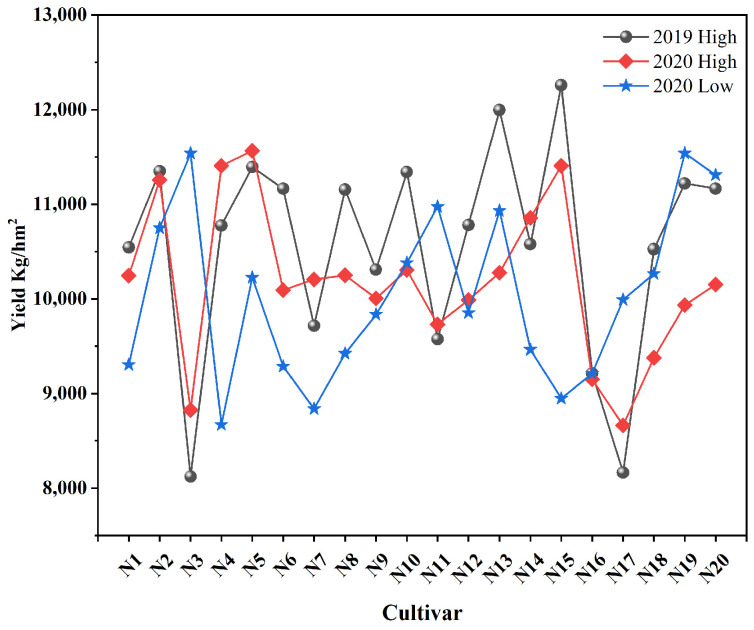
Comparison of yield trends among the varieties treated with high and low concentrations of nitrogen fertilizers.

The average yields of the high-nitrogen treatment in 2019 and 2020 were 10,567.70 kg/hm^2^ and 10,185.03 kg/hm^2^, respectively, and the average yield of the low-nitrogen treatment in 2020 was 10,037.22 kg/hm^2^ (Figure 1). The yield of the high-nitrogen treatment in 2019 was significantly higher than that of the high- and low-nitrogen treatments in 2020; however, the difference between the high- and low-nitrogen treatment yields in 2020 was not significant. Moreover, the change trend of the yield between high-nitrogen treatment varieties in 2019 and 2020 was the same. However, the changes between the low-nitrogen treatment varieties in 2020 were more complicated.

**Figure 2:**
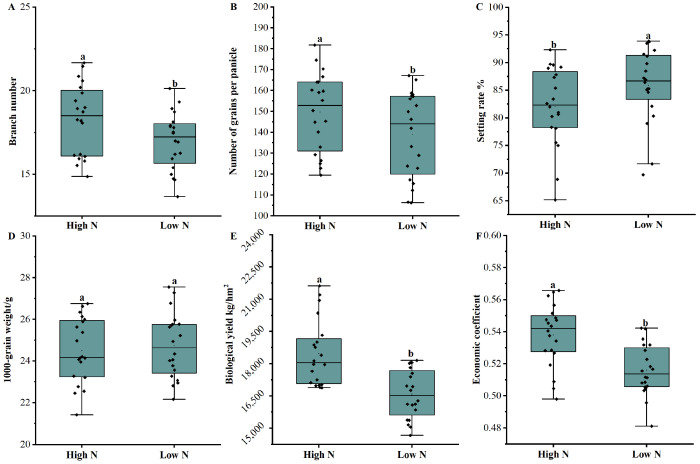
Comparison of the yield components between the high- and low-nitrogen (N) fertilizer treatments. (**A**): Branch number; (**B**): number of grains per panicle; (**C**): setting rate; (**D**): 1000-grain weight; (**E**): biological yield; and (**F**): economic coefficient. Vertical lines represent the standard deviation of the measurements. Values marked by the same letter do not differ according to Tukey’s honestly significant difference at a 5% confidence level.

The analysis of the yield components revealed that the tiller number and grain numbers per panicle in the high-nitrogen treatment group was significantly higher than those in the low-nitrogen treatment group, whereas the grain-setting rate was significantly lower than that in the low-nitrogen treatment group. However, no significant difference was observed in the 1000-grain weight between the high- and low-nitrogen treatment groups (Figure 2). Moreover, the biological yield of the high-nitrogen treatment group was significantly higher than that of the low-nitrogen treatment group, and the economic coefficient of the high-nitrogen treatment group was significantly lower than that of the low-nitrogen treatment group.

Based on the high-nitrogen treatment output, the test materials were divided into high-yield (seven varieties with an output > 9900 kg/hm^2^ and seven varieties with a yield of 9100–9900 kg/hm^2^) and low-yield groups (six varieties with a yield < 9100 kg/hm^2^). According to the low-nitrogen treatment taste value, the test materials were divided into high-taste (seven varieties with a taste value > 66), intermediate-taste (seven varieties with a taste value of 61–66), and low-taste (six varieties with a taste value < 61) groups.

Table 1 shows the variation trend in yield structure for three different yield levels under high- and low-nitrogen treatments. Under the high-nitrogen treatment, the panicle number of the high-yield group was greater than that of the intermediate- and low-yield groups. However, under the low-nitrogen treatment, the panicle numbers of the high- and intermediate-yield groups were greater than that of the low-yield group. Moreover, we observed no significant differences in the total number of grains per panicle among the high-, intermediate-, and low-yield groups under the high-nitrogen treatment; however, the number of grains per panicle of the intermediate-yield group was greater than that of the high- and low-yield groups under the low-nitrogen treatment.

### 2.2. Quality and Panicle Traits

**Figure 3:**
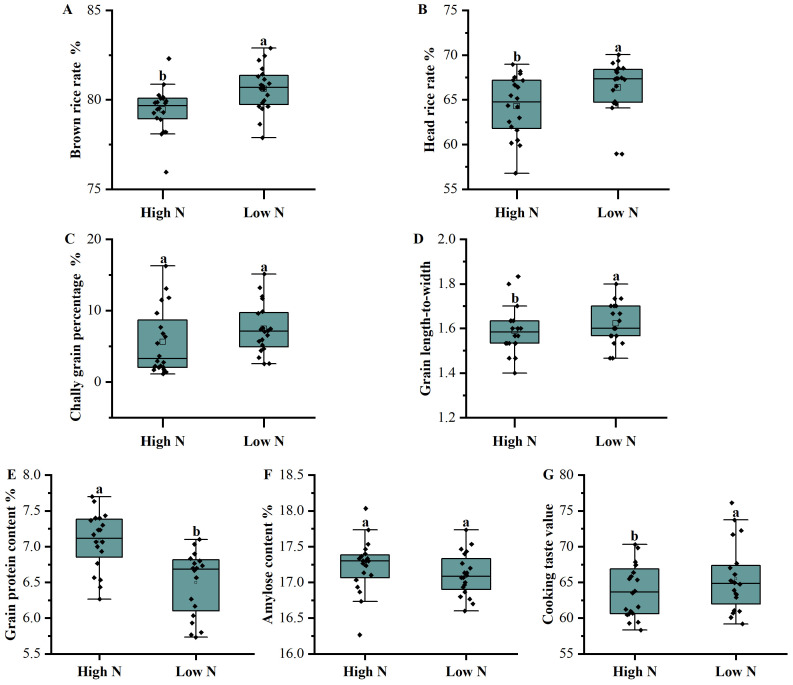
Comparison of the quality traits between the high- and low-nitrogen (N) fertilizer treatments. (**A**): Brown rice rate; (**B**): head rice rate; (**C**): chalky rice percentage; (**D**): grain length-to-width ratio; (**E**): grain protein content; (**F**): amylose content; and (**G**): cooking taste value. Values marked by the same letter do not differ according to Tukey’s honestly significant difference at a 5% confidence level.

Figure 3 shows the comprehensive analysis results of the quality traits. The brown and polished rice rates under the low-nitrogen treatment were higher than those under the high-nitrogen treatment. Additionally, the chalk-white grain rate and grain aspect ratios under the low-nitrogen treatment were significantly higher than those under the high-nitrogen treatment. The protein content of the low-nitrogen treatment group was significantly lower than that of the high-nitrogen treatment group. Lastly, the taste value in the low-nitrogen treatment group was significantly higher than that in the high-nitrogen treatment group, and the amylose content did not differ significantly between the high- and low-nitrogen treatments.

For the primary branch, we observed no significant difference between the branch numbers of the low- and high-nitrogen treatment groups. Additionally, the setting rate and 1000-grain weight of the primary branch under the high-nitrogen treatment were significantly lower than those under the low-nitrogen treatment (Table 2). For the secondary branch, the branch number and peduncles in the high-nitrogen treatment group were significantly higher than those in the low-nitrogen treatment group. Additionally, the setting rate under the high-nitrogen treatment was significantly lower than that under the low-nitrogen treatment. However, no significant difference in the 1000-grain weight was observed between the high- and low-nitrogen treatments. Finally, the panicle length, grain density, and panicle type index of the high-nitrogen treatment group were significantly higher than those of the low-nitrogen treatment group (Table 3).

### 2.3. Relationship between the Yield and Quality Traits

**Figure 4:**
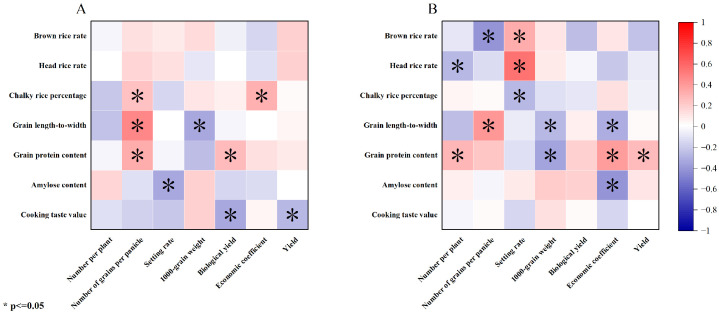
Relationship between the yield and quality traits. (**A**). High-nitrogen treatment. (**B**). Low-nitrogen treatment. Yield traits: number per plant; number of grains per panicle; setting rate; 1000-grain weight; biological yield; economic coefficient; and yield. Quality traits: brown rice rate; head rice rate; chalky rice percentage; grain length-to-width ratio; grain protein content; amylose content; and cooking taste value.

The grain rate of brown rice treated with a high concentration of nitrogen fertilizer was negatively correlated with the yield, and the rate of polished rice was not significantly correlated with the yield traits, similar to that observed under the low-nitrogen treatment. Moreover, the chalk-white grain rate was significantly positively correlated with the panicle number and economic coefficient. The grain aspect ratio was significantly positively correlated with the panicle number and negatively correlated with the 1000-grain weight. Furthermore, the protein content was significantly positively correlated with the panicle grain number and biological yield. Lastly, the amylose content was only negatively correlated with the setting rate, and the taste value was significantly negatively correlated with the biological yield and grain yield (Figure 4A).

The correlation analysis showed that the brown rice rate under the low-nitrogen treatment was inversely correlated with the number of grains per panicle and positively correlated with the setting rate. Meanwhile, the polished rice rate was not significantly correlated with the yield traits. Additionally, the chalk-white grain rate was significantly and negatively correlated with the setting rate and positively correlated with the economic coefficient. Moreover, the grain aspect ratio was significantly positively correlated with the panicle number and negatively correlated with the economic coefficient. The protein content was significantly positively correlated with the number of panicles, economic coefficients, and yield and negatively correlated with the 1000-grain weight. Additionally, the amylose content was only negatively correlated with the economic coefficient (Figure 4B).

Table 3 presents the following trends. The brown rice rates of the high- and low-yield groups were significantly higher than those of the low-yield group under the high-nitrogen treatment; however, no significant difference was observed under the low-nitrogen treatment. Additionally, the polished rice rate of the low-yield group was significantly higher than that of the high- and intermediate-class groups under the high- and low-nitrogen treatments. The chalk particle rate of the high-yield group was significantly lower than that of the intermediate- and low-yield groups under the high-nitrogen treatment; however, no significant difference was observed among the groups under the low-nitrogen treatment. The grain aspect ratio of the low-yield group was higher than that of the high- and intermediate-yield groups under the low-nitrogen treatment. The high- and intermediate-yield groups had significantly higher protein contents than the low-yield group under the high-nitrogen treatment; however, no significant difference was observed among the groups under the low-nitrogen treatment. Furthermore, no significant differences in the amylose contents existed between the treatment groups under the low- and high-nitrogen treatments. The taste value of the low-yield group was significantly higher than that of the high- and intermediate-yield groups under the high-nitrogen treatment. However, the high-yield group had a significantly lower taste value than the intermediate- and low-yield groups under the low-nitrogen treatment.

### 2.4. Relationship among the Yield, Quality, and Panicle Traits

**Figure 5:**
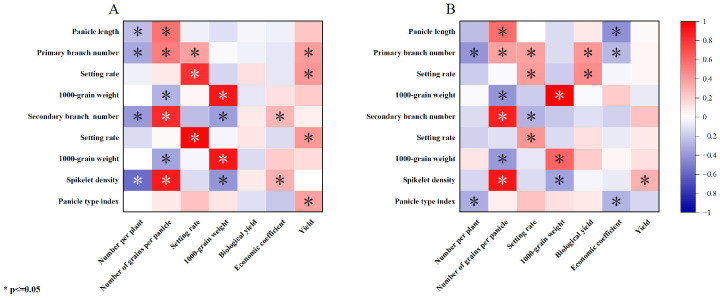
Relationship between the panicle and yield traits. (**A**). High-nitrogen treatment. (**B**). Low-nitrogen treatment. Yield traits: number per plant; number of grains per panicle; setting rate; 1000-grain weight; biological yield; economic coefficient; and yield. Panicle traits: panicle length; primary branch number; setting rate; 1000-grain weight; secondary branch number; setting rate; spikelet density; and panicle type index.

The correlation of the panicle traits with the composition factors of the high-nitrogen treatment was similar to that with composition factors of the low-nitrogen treatment; however, the biological yield was not closely related to the panicle traits. The economic coefficient was positively correlated with the particle density and secondary stem number, and the yield was significantly positively correlated with the panicle index, primary and secondary stem-setting rate, and primary stem number (Figure 5A). The relationship between the yield and panicle traits was complex; the number of panicles treated with a low-nitrogen concentration was significantly negatively correlated with the panicle type index and number of primary branches. Additionally, the number of grains was positively correlated with the particle density, number of primary and secondary peduncles, and panicle length and negatively correlated with the 1000-grain weight of primary and secondary peduncles. Furthermore, the setting rate was positively correlated with the setting rate of primary and secondary peduncles and the number of primary peduncles and negatively correlated with the number of secondary peduncles. The 1000-grain weight was negatively correlated with the grain density and positively correlated with the 1000-grain weight of the primary and secondary peduncles. The biological yield was significantly positively correlated with the number of primary branches and the setting rate. Furthermore, the economic coefficient was negatively correlated with the panicle index, panicle length, and number of primary peduncles, and the yield was only positively correlated with the grain density (Figure 5B).

As presented in Table 4, for the primary branch, the primary branch numbers per panicle of the high-yield group receiving the high-nitrogen treatment was significantly lower than that of the intermediate- and low-yield groups. The primary branch numbers per panicle of the high-yield group receiving the low-nitrogen treatment was significantly lower than that of the low-yield group. Additionally, the setting rate of the high-yield group treated with a high concentration of nitrogen fertilizer was significantly higher than that of the intermediate- and low-yield groups. However, no significant difference was observed between all the yield types under the low-nitrogen treatment. The 1000-grain weight of the low-yield group treated with high-nitrogen fertilizer concentrations was significantly lower than that of the high- and intermediate-yield groups. Additionally, the 1000-grain weight of the intermediate-yield group treated with the low-nitrogen fertilizer was significantly higher than that of the high- and low-yield groups.

For the secondary branch, under the high-nitrogen treatment, the stem number of the high-yield group was significantly higher than that of the intermediate- and low-yield groups. Under the low-nitrogen treatment, the stem number of the high- and intermediate-yield groups was significantly higher than that of the low-yield group. Additionally, the setting rate and 1000-grain weight of the high-yield groups under the high- and low-nitrogen treatments were significantly higher than those of the intermediate- and low-yield groups.

Under the high-nitrogen treatment, the panicle length of the high-yield groups was significantly higher than that of the intermediate- and low-yield groups. Additionally, the panicle length of the high- and intermediate-yield groups under the low-nitrogen treatment was significantly higher than that of the low-yield group. The particle density of the high-yield group treated with a low nitrogen concentration was significantly higher than that of the intermediate- and low-yield groups. Lastly, the panicle type index of the intermediate-yield group under the high-nitrogen treatment was significantly higher than that of the high- and low-yield groups. However, under the low-nitrogen treatment, the panicle type index of the high-yield group was significantly higher than that of the intermediate- and low-yield groups.

**Figure 6:**
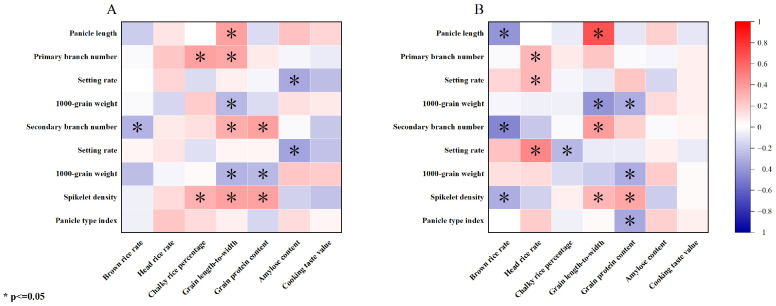
Relationship between the quality and panicle traits. (**A**). High-nitrogen treatment. (**B**). Low-nitrogen treatment. Quality traits: brown rice rate; head rice rate; chalky rice percentage; grain length-to-width ratio; grain protein content; amylose content; and cooking taste value. Panicle traits: panicle length; primary branch number; setting rate; 1000-grain weight; secondary branch number; setting rate; spikelet density; and panicle type index.

Under the high-nitrogen treatment, the brown and polished rice rates were negatively and positively correlated with the number of secondary stems and panicle length, respectively. Additionally, the chalk-white grain rate was significantly and positively correlated with the grain density and the number of primary branches. Furthermore, the grain aspect ratio was positively correlated with the grain density and the number of primary and secondary branches. The panicle length was negatively correlated with the 1000-grain weight of the primary and secondary branches. In turn, the protein content was significantly and positively correlated with the grain density and the number of secondary branches and negatively correlated with the 1000-grain weight of the secondary branches. Furthermore, the amylose content was significantly and negatively correlated with the setting rate of the primary and secondary branches. Lastly, the correlation between the taste values and panicle traits was not significant (Figure 6A).

The correlation analysis results (Figure 6) revealed that the brown rice rate was significantly and negatively correlated with the grain density, secondary stem number, and panicle length under the low-nitrogen treatment. Additionally, the polished rice rate was positively correlated with the setting rate of the primary and secondary branches and the number of primary branches; meanwhile, the chalk-white grain rate was negatively correlated with the setting rate of the secondary branches. The grain aspect ratio was positively correlated with the grain density, secondary stem number, and panicle length but negatively correlated with the 1000-grain weight of the primary branches. Additionally, the protein content was significantly and positively correlated with the grain density and negatively correlated with the panicle type index, the primary and secondary stem, and the 1000-grain weight. Lastly, the amylose content and taste values were not significantly correlated with the panicle traits (Figure 6B).

### 2.5. Relationship among the Taste, Yield, and Quality Traits

Certain differences existed in the yield traits of the different flavor groups (Table 5). Under the high-nitrogen treatment, the panicle number of the low-taste group was significantly higher than that of the high- or intermediate-taste groups; however, no significant difference in the panicle number was observed under the low-nitrogen treatment. The number of panicle grains was significantly higher in the intermediate-taste group under the high-nitrogen treatment than in the high- or low-taste groups; however, under the low-nitrogen treatment, no significant difference was observed among the taste groups. Furthermore, the setting rate was significantly higher under the low-nitrogen treatment in the high-taste group than in the intermediate- or low-taste groups. No significant difference was observed for the 1000-grain weight among all taste groups under the high-nitrogen treatment. However, under the low-nitrogen treatment, the 1000-grain weight of the high-taste group was significantly higher than that of the intermediate- or low-taste groups. Lastly, the yield of the high-taste group was significantly lower than that of the intermediate- or low-taste groups under the low-nitrogen treatment.

The further comparison of the quality traits among the different taste types (Table 6) showed no significant differences in the brown rice rates among all taste groups under the low-nitrogen treatment. Under the high-nitrogen treatment, the polished rice rate of the intermediate-flavor group was significantly higher than that of the high- or low-taste groups. However, under the low-nitrogen treatment, the polished rice rate of the high- and intermediate-taste groups was significantly higher than that of the low-taste group. Under the high-nitrogen treatment, the chalk-white grain rate of the low-taste group was significantly higher than that of the high- or intermediate-taste groups. However, under the low-nitrogen treatment, the chalk-white grain rate of the intermediate-taste group was significantly higher than that of the high- or low-taste groups. Furthermore, the grain aspect ratio was significantly lower under the low-nitrogen treatment in the high-taste group than in the intermediate- or low-taste groups. The protein content in the low-taste group was significantly higher than that in the high-taste group under the low-nitrogen treatment. Lastly, we observed no significant difference in the amylose content among all taste groups under the low-nitrogen treatment.

As presented in Table 7, the number of primary branches in the intermediate- and low-taste groups was significantly higher than that in the high- and intermediate-taste groups under the low-nitrogen treatment; however, no significant difference was observed under the high-nitrogen treatment. Under the low-nitrogen treatment, the seed-setting rates of the intermediate- and high-taste groups were significantly higher than those of the low-taste groups, and the 1000-grain weight of the high-taste group was significantly higher than that of the low-taste group. The number of secondary branches in the high-taste group was significantly lower than that in the intermediate- and low-taste groups under high-nitrogen treatment. The seed-setting rate and 1000-grain weight of the intermediate- and high-taste groups were significantly higher than those of the low-taste groups under both treatments. Under the high-nitrogen treatment, the panicle length of the high-taste group was significantly higher than that of the low-taste group, and the grain density of the high-taste group was significantly lower than that of the low-taste group. The panicle type index was significantly higher under both treatments than in the low-taste group.

## 3. Discussion

### 3.1. High-Yield and High-Quality Approach for Erect-Panicle Japonica Rice Breeding in Northern China

In the 1980s, the breeding and promotion of erect-panicle-type Liaojing No. 5 played a key role in improving the yield potential of northern japonica rice. Afterward, the cultivation of erect-panicle-type japonica rice varieties significantly improved the fruitfulness and quality of northern japonica rice, consistent with the goal of maintaining a high-yield potential, which became an important feature and innovation point of japonica rice breeding in China [1]. At the beginning of the 21st century, the yield of japonica rice in northeastern China increased by 16.60%, significantly more than the national average increase of 14.76% in the same period [2]. In our study, the average yield following the high- and low-nitrogen treatments exceeded 10,000.00 kg/hm^2^ (666.67 kg/mu), reaching a maximum of 12,285.63 kg/hm^2^ (817.24 kg/mu). According to the national standard for high-quality rice (GB/T17891-2017) [8], the polished rice rate reached grade 3 in the main quality indicators; however, chalk whiteness did not reach the grade 3 standard. Moreover, the high-yield varieties under the high-nitrogen treatment were characterized by significant increases in panicle number, grain-setting rate, 1000-grain weight, the number of secondary stems, secondary stem-setting rate, secondary peduncle 1000-grain weight, grain density, and protein content. However, the brown rice, polished rice, and chalk-white grain rates and taste values were significantly reduced. In addition to its significant negative correlation with taste value, the yield of the rice varieties was not closely related to other major quality traits. Therefore, future research should focus on increasing the polished rice rate, reducing chalk whiteness and protein content, and improving the taste quality of northern erect-panicle-type japonica rice to the level of traditional japonica rice while maintaining its high yield. In the past, the quality of japonica rice was significantly better than that of indica rice. The quality of indica rice has recently improved; however, the difference in quality between indica and japonica rice remains large [9]. Introducing the indica rice lineage is expected to increase the japonica rice yield potential; however, its effects on quality warrant further study [10].

### 3.2. Efficient Nitrogen Utilization by Northern Erect-Panicle Japonica Rice

The erect-panicle type (regulated by *DEP1*) is another important morphological improvement in japonica rice to adapt to the high-yield demand after dwarf breeding using the semi-dwarf stalk gene (*SD1*) [2]. Related studies have explained the genetic, physiological, and ecological mechanisms underlying the yield increase induced by the key gene *DEP1* [11]. *DEP1* is reportedly directly related to nitrogen use efficiency [12]. The amount of fertilization required to maximize the yield potential, especially excessive nitrogen fertilizer, has become a common problem in cultivating erect-panicle japonica rice varieties in China [13]. Our study revealed that the 20 tested varieties could be divided into four types: (1) high-nitrogen–high-yield and low-nitrogen–high-yield, including No. 2 Beijing 2, No. 10 Liaojing 212, No. 13 Tie Japonica 20, and No. 19 Fuhe 5; (2) high-nitrogen–high-yield and low-nitrogen–low-yield, including No. 1 Beijing 1, No. 4 Beijing 1501, No. 5 Beijing 1604, No. 6 Shendao 47, No. 8 Liaojing 401, No. 12 Tiejing 11, No. 14 Jindao 108, and No. 15 Jindao 201; (3) high-nitrogen–low-yield and low-nitrogen–high-yield, including No. 3 North Japonica 3, No. 11 Tiejing 7, No. 18 Yanjing 662, and No. 20 Meifeng Rice 669; and (4) high-nitrogen–low-yield and low-nitrogen–low-yield, including No. 7 Liaojing 1415, No. 9 Liaojing 399, No. 16 Jindao 104, and No. 17 Yanjing 765. From a breeding and production perspective, nitrogen-efficient varieties should have high yields, regardless of the nitrogen fertilization level applied [9]. The comprehensive analysis of the literature and the results of our study show the need and feasibility of cultivating rice varieties with stable and high yields under different nitrogen levels. The nitrogen use efficiency of indica rice is generally higher than that of japonica rice [14]. Therefore, searching for an effective interaction between DEP1 and the indica genome and related genes may be useful for achieving nitrogen efficiency in northern erect-panicle rice.

### 3.3. Improvement in the Taste Quality of Northern Erect-Spike-Type Japonica Rice

Food quality is a comprehensive index of the appearance and improvement in taste quality of northern erect-panicle-type japonica rice and includes smell, taste, hardness, and viscosity of rice and a comprehensive reflection of appearance, smell, taste, and touch when eating, which makes it the most complex quantitative trait in rice. Factors affecting taste quality are complex, among which the amylose and protein contents play key roles. Reducing the amylose and protein contents within a certain range is conducive to improving taste quality [15]. Compared with Japanese japonica rice, northern China has many japonica rice varieties with low amylose and protein contents (amylose content < 16.70% and protein content < 6.24%) [16]. In this study, the grain amylose content under the high- and low-nitrogen treatments exceeded 17.0%, and the protein content was >6.5% after the low-nitrogen treatment and 7.0% after the high-nitrogen treatment. Therefore, reducing the amylose and protein contents may be an effective way to improve the taste quality of northern erect-panicle japonica rice. Amylopectin accounts for approximately 80% of endosperm starch. Considering amylose and cultivars with a similar protein content, the length distribution of the amylopectin chain is the main factor affecting the structure and gelatinization characteristics of starch, and increasing the ratio of short-chain amylopectin will improve taste quality [13,15]. In a previous study, the high temperatures during the grain-filling period of japonica rice in southern Jiangsu increased the long-chain ratio of amylopectin, thereby reducing the amylose content, promoting nitrogen absorption, and improving the protein content, which in turn lowered the rice quality, particularly the taste quality. Later, the use of the Wxmp gene to reduce the amylose content was adopted to improve the taste quality and achieved significant results [15,17]. Comparatively, in another study, the climatic conditions at the northern japonica rice-filling stage were better than those at the southern Jiangsu and Zhejiang rice-filling stages; northern japonica rice grains had higher amylose contents, lower protein contents, and a better overall taste quality than Jiangsu japonica rice [18]. Furthermore, dep1 is a gain-of-function mutation that regulates multiple phenotypes by encoding G protein γ subunits [19]. Our research group found no significant differences in the amylose and protein contents between *DEP1*/*DEP1* and *dep1*/*dep1*; however, the taste value of *DEP1*/*DEP1* was significantly higher than that of *dep1*/*dep1* (unpublished results from Shenyang Agricultural University). Therefore, elucidating the molecular mechanism by which DEP1 affects the taste quality via the long distribution of pullulan chains could provide a new approach for improving the taste quality of northern erect-panicle japonica rice.

Recently, food quality has received increasing attention, and there is an endless stream of taste evaluation activities worldwide [2]. First, from a possibility analysis perspective, Japan’s famous rice variety Koshihikari was bred by the Fukui Prefecture Agricultural Experimental Farm and the Niigata Prefecture Agricultural Experimental Field and hybridized from 1946 to its official name in 1956, during which Japan’s post-World War II recovery period experienced a food supply shift from extreme shortage to basic demand [20]. Therefore, quality, especially taste, cannot be the main breeding goal; a good taste may only be a probable phenomenon in the breeding process. For nearly 70 years, scientists have been trying to make rice varieties that taste better than Koshihikari; however, in addition to the significant improvements in disease and lodging resistance, no varieties that significantly exceed the Koshihikari taste have been bred. Second, from a necessity analysis perspective, rice is crystal clear, fragrant, sweet, soft, and silky, and the indicators of appearance, smell, taste, hardness, viscosity, and rice retrogradation are all in the appropriate range of organic combinations. However, it is unclear which index has obvious deficiencies and needs improvement. Thus, from a long-term perspective, the primary objective of enhancing the quality of northern erect-panicle japonica rice should be centered around improving processing and appearance characteristics while aiming to match them with the taste quality of ordinary Japanese japonica rice.

## 4. Materials and Methods

### 4.1. Plant Cultivation and Harvesting

Twenty erect-panicle-type japonica rice varieties recently bred in Liaoning Province were used as the test materials (Table 8). They were planted in 2019 and 2020 at the Liaozhong Kalima Rice Experimental Station of Shenyang Agricultural University. The seeds were sown on 27 April 2019 and 26 April 2020 and transplanted on 28 May 2019, and 26 May 2020. The test setup comprised two nitrogen treatments: high nitrogen: 225 kg/hm^2^ and low nitrogen: 150 kg/hm^2^; P_2_O_5_ 70 kg/hm^2^; and K_2_O 180 kg/hm^2^. In the high-nitrogen treatment area, the nitrogen fertilizer (base fertilizer): tillering fertilizer: ear fertilizer: grain fertilizer ratio was 2.7:4.6:2.3:1.4; in the low-nitrogen treatment area, the nitrogen fertilizer (base fertilizer): tillering fertilizer: panicle fertilizer: grain fertilizer ratio was 2.7:3.7:2.3:1.4. The ratio of potassium fertilizer (base fertilizer): tiller fertilizer: panicle fertilizer was 3.0:3.0:6.0, and all phosphate fertilizers were applied as base fertilizers. Furthermore, we adopted a split-zone design, with the nitrogen application rate as the main zone and the variety as the secondary zone. This was repeated thrice. Each variety was planted in 10 rows with 20 plants per row, a row spacing of 30 cm, and a plant spacing of 16.5 cm. The biological yield (the total crop dry matter harvested per unit of land) and economic coefficient (the ratio of the grain yield of a crop to the biological yield) were measured.

In each area during the mature period, the length of the long position was neat and even, and the natural wind was suspended using a net. To investigate the effective panicle number, 10 panicles were selected according to the number of primary branches. Additionally, we investigated the panicle length, number of grains per panicle, number of primary branches, number of secondary stems, primary stemming rate, secondary stemming rate, panicle type index (the ratio of the number of primary branches to the cob node position where the number of primary branches was largest), and the 1000-grain weights of the primary and secondary stems. All communities were harvested, threshed, and tested.

The harvested rice was stored at room temperature (10 °C) for 3 months, and the quality traits were determined according to the national standard GB/T 15682-2008 [21] Grain and Oil Inspection Sensory Evaluation Method for Cooking and Edible Quality of Rice.

### 4.2. Determination of the Processing Quality

The brown rice rate was measured using an FC2K brown rice machine (Qiushan Technology (Dongguan) Co., Ltd., Shenzhen, Guangdong, China), and the precision and polishing rates were determined using a VP-32 rice-polishing machine (Yamamoto, Yamamoto Co., Ltd., Tokyo, Japan).

### 4.3. Determination of the Appearance Quality

A GR/JMWT12 rice appearance tester (Beijing Dongfu Jiuheng Instrument Technology Co., Ltd., Beijing, China) was used to determine the chalk-white grain rate, chalk whiteness, and grain type (aspect ratio).

### 4.4. Determination of the Nutrition Quality

The protein and amylose contents of rice were determined using an Infratec TM 1241 Grain Analyzer (Foss GmbH, Hamburg, Germany).

### 4.5. Determination of the Taste Quality

The rice taste value was determined using an STA-1A flavor meter (Satake Corporation, Tokyo, Japan). We used a rice taste analyzer to determine the taste value of rice. After weighing 30 g of rice, we washed it with water within 30 s and placed it in special stain-less steel tank to ensure that the ratio of rice to water was 1:1.35 (indica rice was 1:1.4 and japonica rice was 1:1.35). Then, we soaked it for 30 min, steamed it in a rice cooker for 30 min, and kept it warm for 10 min. Finally, we placed it at room temperature for 90 min to determine the taste value of the rice, including hardness, stickiness, balance value, and taste value. A high taste value often indicates a better taste quality.

### 4.6. Statistical Analysis

MS Excel 2016 (Microsoft Corp., Redmond, WA, USA), IBM SPSS Statistics 29 (IBM Corp., Armonk, NY, USA), and Origin 2021 (Origin Lab, Northampton, MA, USA) were used for statistical analysis and graphing. Duncan’s multiple comparison method was used to assess the significance of differences (α = 0.05). The results for Nipponbare mutants were analyzed using a one-way analysis of variance, followed by Dunnett’s multiple comparison test [22]. The statistical method used to compare between groups was a two-way analysis of variance (*p* < 0.05).

## 5. Conclusions

The average yield following the high- and low-nitrogen treatments exceeded 10,000.00 kg/hm^2^, with a maximum of 12,285.63 kg/hm^2^. The high-nitrogen treatment significantly increased the number of panicles and grains, decreasing the percentage of filled grains. Additionally, the high-nitrogen treatment significantly increased the biomass and decreased the economic coefficient. The taste value was significantly negatively correlated with the biological and grain yields, with no significant correlation with the panicle characteristics. The high-yield–high-nitrogen treatment group had more panicles, a higher seed-setting rate, and a higher 1000-grain weight than the other groups. The high-yield–low-nitrogen group had a higher number of panicles and seed-setting rate than the other groups. The low-yield–high-nitrogen groups had lower whole grain, grain length-to-width ratio, and taste values than the other groups. The low-yield–low-nitrogen group had fewer primary branches than the other groups; excluding the primary branch-setting rate and 1000-grain weight, the other panicle traits of the group were significantly higher than those of the other groups. The high-nitrogen–high-flavor group had lower panicle and spikelet numbers and higher spikelet fertility rates than the other groups. The low-nitrogen–high-flavor group had higher spikelet fertility rates and 1000-grain weight than the other groups. Compared to the other groups, the low-nitrogen–high-flavor group had a higher head rice yield, and the high-nitrogen–high flavor group had a lower chalkiness rate. The main goal of the breeding and cultivation of high-yield and high-quality erect-panicle japonica rice in northern China is to achieve “dual high, dual low, and one high and one low” conditions, signifying a high yield with high- or low-nitrogen levels, low protein and amylose contents, high head rice rates, and low chalkiness. This study provides a new technique for enhancing the taste of northern erect-panicle japonica rice to enhance the sustainable, high-yield, and high-quality development of japonica rice in northern China.

The future breeding direction of japonica rice in northern China should be to breed japonica rice varieties with a high nitrogen use efficiency, high yield, and a low protein content and amylose content, that is, double high and double low type varieties. Such rice varieties tend to have the following characteristics: low chalky grain rate, high yield, good starch quality, high filling rate, high nitrogen use efficiency, good taste, and good rice quality. Combined with scientific cultivation methods, the input of nitrogen fertilizer was reduced to achieve a high yield and high quality.

## Figures and Tables

**Table 1:** Comparison of the yield components among the three yield levels under high- and low-nitrogen treatments.

Trait	Fertilizer Model	High Yield	Intermediate Yield	Low Yield
Panicle number	High	20.01 ± 3.11 a	16.71 ± 3.32 b	16.09 ± 3.85 b
	Low	16.62 ± 3.21 a	16.39 ± 3.14 a	14.79 ± 2.79 b
Number of grains per panicle	High	152.43 ± 10.68 a	153.09 ± 7.88 a	148.59 ± 8.87 a
	Low	130.81 ± 8.46 b	148.13 ± 7.79 a	135.25 ± 8.54 b
Setting rate (%)	High	84.62 ± 2.62 a	82.55 ± 2.52 b	82.28 ± 2.33 b
	Low	86.32 ± 1.53 a	84.63 ± 1.51 b	86.28 ± 1.21 a
1000-grain weight (g)	High	25.69 ± 2.78 a	25.01 ± 1.97 b	23.58 ± 1.41 b
	Low	25.40 ± 3.21 a	25.34 ± 3.14 a	23.68 ± 2.72 b
Grain yield kg/hm^2^	High	10,468.22 ± 331.88 a	9413.93 ± 295.93 b	8669.67 ± 263.32 c
	Low	8472.01 ± 299.47 a	8312.89 ± 370.71 a	8048.26 ± 277.86 b

Values marked by the same letter do not differ according to Tukey’s honestly significant difference at a 5% confidence level.

**Table 2:** Comparison of the panicle traits between the high- and low-nitrogen fertilizer treatments.

Traits		High Nitrogen	Low Nitrogen
Primary branch	Branch number	11.20 ± 1.16 a	12.42 ± 1.13 a
	Setting rate (%)	93.54 ± 0.23 b	95.45 ± 0.22 a
	1000-grain weight (g)	25.75 ± 1.51 b	26.51 ± 1.69 a
Secondary branch	Branch number	27.40 ± 4.93 a	25.31 ± 6.24 b
	Setting rate (%)	72.97 ± 0.12 b	77.64 ± 0.12 a
	1000-grain weight (g)	22.93 ± 1.88 a	23.31 ± 1.62 a
Panicle length (cm)		17.13 ± 0.82 a	16.87 ± 0.99 b
Spikelet density/10 cm		86.91 ± 9.72 a	83.86 ± 11.52 b

Values marked by the same letter do not differ according to Tukey’s honestly significant difference at a 5% confidence level.

**Table 3:** Comparison of the quality traits among yield levels under high- and low-nitrogen treatments.

Traits	Fertilizer Model	High Yield	Intermediate Yield	Low Yield
Brown rice rate (%)	High	76.53 ± 3.40 a	75.44 ± 2.87 a	73.98 ± 2.18 b
	Low	80.49 ± 1.33 a	78.73 ± 1.65 a	78.24 ± 1.02 a
Head rice rate (%)	High	52.72 ± 0.99 c	57.27 ± 1.08 b	58.48 ± 1.11 a
	Low	60.85 ± 1.14 b	57.13 ± 1.07 c	61.55 ± 1.16 a
Chalky rice percentage (%)	High	25.05 ± 3.19 b	26.06 ± 4.15 a	26.17 ± 3.92 a
	Low	27.78 ± 3.59 a	27.33 ± 4.94 a	27.87 ± 4.83 a
Grain length-to-width ratio	High	1.62 ± 0.03 b	1.62 ± 0.02 b	1.66 ± 0.04 a
	Low	1.61 ± 0.03 b	1.62 ± 0.05 b	1.68 ± 0.03 a
Grain protein content (%)	High	7.29 ± 0.02 a	7.28 ± 0.05 a	7.04 ± 0.07 b
	Low	6.88 ± 0.09 a	6.61 ± 0.12 a	6.46 ± 0.16 a
Amylose content (%)	High	17.23 ± 0.49 a	16.81 ± 0.37 a	16.91 ± 0.37 a
	Low	16.44 ± 0.18 a	15.98 ± 0.38 a	16.12 ± 0.38 a
Cooking taste value	High	62.71 ± 1.22 b	65.76 ± 1.20 b	65.4 ± 1.17 a
	Low	64.18 ± 1.21 b	67.51 ± 1.23 a	66.35 ± 1.19 a

Values marked by the same letter do not differ according to Tukey’s honestly significant difference at a 5% confidence level.

**Table 4:** Comparison of the panicle traits at different yield levels under the high- and low-nitrogen treatments.

Trait		Fertilizer Model	High Yield	Intermediate Yield	Low Yield
Primary branch	Branch number	High	11.88 ± 3.36 b	12.51 ± 2.38 a	12.86 ± 3.39 a
		Low	11.64 ± 2.35 b	12.21 ± 2.01 ab	12.59 ± 2.48 a
	Setting rate (%)	High	96.26 ± 3.36 a	94.75 ± 4.39 b	94.40 ± 4.36 b
		Low	96.25 ± 4.44 a	95.11 ± 3.59 a	93.83 ± 4.01 a
	1000-grain weight (g)	High	26.52 ± 1.77 a	26.63 ± 1.68 a	24.89 ± 1.58 b
		Low	26.28 ± 1.86 b	27.42 ± 1.63 a	25.59 ± 1.78 b
Secondary branch	Branch number	High	30.06 ± 4.91 a	26.48 ± 3.84 b	25.91 ± 2.79 b
		Low	26.27 ± 3.80 a	26.39 ± 3.68 a	23.39 ± 3.21 b
	Setting rate (%)	High	74.09 ± 2.25 a	70.07 ± 2.83 b	70.42 ± 2.15 b
		Low	77.3 ± 3.35 a	74.72 ± 2.94 b	75.04 ± 2.28 b
	1000-grain weight (g)	High	23.59 ± 0.92 a	22.72 ± 0.69 b	22.32 ± 0.38 b
		Low	24.89 ± 0.96 a	22.96 ± 0.78 b	21.73 ± 0.66 b
Panicle length (cm)		High	17.67 ± 2.11 a	16.7 ± 3.15 b	16.09 ± 3.35 b
		Low	16.62 ± 1.21 a	16.39 ± 1.14 a	14.79 ± 2.29 b
Spikelet density/10 cm		High	89.98 ± 8.73 a	84.44 ± 5.69 b	82.55 ± 6.51 b
		Low	84.53 ± 4.69 a	80.46 ± 4.57 b	78.23 ± 3.38 b
Panicle type index		High	0.45 ± 0.01 b	0.47 ± 0.01 a	0.45 ± 0.01 b
		Low	0.47 ± 0.01 a	0.43 ± 0.01 c	0.46 ± 0.01 b

Values marked by the same letter do not differ according to Tukey’s honestly significant difference at a 5% confidence level.

**Table 5:** Comparison of the yield traits among the different taste groups in a population of 20 rice varieties grown under high- and low-nitrogen treatments.

Traits	Fertilizer Model	High Cooking Taste	Intermediate Cooking Taste	Low Cooking Taste
Panicle number	High	15.93 ± 3.34 b	16.28 ± 2.41 b	17.6 ± 1.93 a
	Low	15.65 ± 1.41 a	15.55 ± 2.33 a	14.97 ± 1.62 a
Number of grains per panicle	High	142.43 ± 12.68 c	153.09 ± 10.88 a	152.59 ± 12.47 b
	Low	130.81 ± 15.46 a	138.13 ± 13.79 a	135.25 ± 12.54 a
Setting rate (%)	High	84.96 ± 2.59 a	81.43 ± 2.53 b	79.85 ± 2.41 c
	Low	89.02 ± 2.49 a	84.05 ± 1.88 b	84.65 ± 1.27 b
Grain weight (g)	High	24.91 ± 1.47 a	24.69 ± 0.99 a	24.47 ± 0.46 a
	Low	25.84 ± 0.88 a	24.38 ± 0.76 b	24.12 ± 0.95 c
Yield kg/hm^2^	High	9483.55 ± 278.42 b	9526.66 ± 289.23 a	9653.6 ± 277.86 a
	Low	8429.31 ± 264.23 b	8652.34 ± 275.95 a	8602.04 ± 265.62 a

Values marked by the same letter do not differ according to Tukey’s honestly significant difference at a 5% confidence level.

**Table 6:** Comparison of the quality traits among the different taste groups in a population of 20 rice varieties grown under high- and low-nitrogen treatments.

Traits	Fertilizer Model	High Cooking Taste	Intermediate Cooking Taste	Low Cooking Taste
Brown rice rate (%)	High	76.68 ± 4.49 a	75.76 ± 1.24 a	73.52 ± 3.66 a
	Low	78.74 ± 3.29 a	81.28 ± 4.49 a	81.13 ± 2.46 a
Polish rice rate (%)	High	54.74 ± 2.66 b	58.67 ± 2.81 a	54.47 ± 1.95 b
	Low	60.95 ± 1.85 a	60.75 ± 1.85 a	57.23 ± 1.74 b
Chalky rice percentage (%)	High	25.12 ± 3.19 b	24.11 ± 4.12 c	26.48 ± 3.23 a
	Low	27.32 ± 3.39 b	28.72 ± 4.41 a	26.92 ± 4.36 b
Grain length-to-width ratio	High	1.54 ± 0.07 c	1.57 ± 0.05 b	1.65 ± 0.10 a
	Low	1.59 ± 0.11 c	1.66 ± 0.05 a	1.63 ± 0.05 b
Grain protein content (%)	High	6.84 ± 0.05 b	7.16 ± 0.13 ab	7.57 ± 0.17 a
	Low	6.57 ± 0.09 b	6.81 ± 0.23 a	6.82 ± 0.18 a
Amylose content (%)	High	16.33 ± 3.62 a	16.22 ± 4.61 a	16.06 ± 3.55 a
	Low	17.71 ± 2.33 a	16.77 ± 4.36 a	16.46 ± 3.38 a
Cooking taste value	High	63.28 ± 0.66 a	63.74 ± 1.67 a	61.26 ± 1.86 b
	Low	70.87 ± 0.71 a	64.32 ± 0.64 b	62.22 ± 0.42 c

Values marked by the same letter do not differ according to Tukey’s honestly significant difference at a 5% confidence level.

**Table 7:** Comparison of the panicle traits among the different taste groups in a population of 20 rice varieties grown under high- and low-nitrogen treatments.

Traits		Fertilizer Model	High Cooking Taste	Intermediate Cooking Taste	Low Cooking Taste
Primary branch	Branch number	High	13.29 ± 1.49 a	12.25 ± 1.23 a	12.36 ± 1.41 a
		Low	12.32 ± 1.17 b	12.37 ± 1.11 b	13.12 ± 1.23 a
	Setting rate (%)	High	92.31 ± 0.28 a	91.70 ± 0.95 a	90.73 ± 0.34 a
		Low	95.83 ± 0.26 a	94.01 ± 0.63 ab	93.04 ± 0.42 b
	1000-grain weight (g)	High	25.84 ± 1.28 a	25.19 ± 1.27 a	24.39 ± 0.61 b
		Low	26.83 ± 0.69 a	26.53 ± 0.52 a	25.25 ± 0.47 b
Secondary branch	Branch number	High	25.83 ± 3.28 b	28.62 ± 3.11 a	28.32 ± 3.32 a
		Low	23.71 ± 2.45 a	24.46 ± 3.38 a	24.64 ± 2.35 a
	Setting rate (%)	High	76.54 ± 1.86 a	72.88 ± 1.37 b	69.41 ± 1.31 b
		Low	82.52 ± 1.71 a	76.22 ± 1.43 b	74.55 ± 1.55 b
	1000-grain weight (g)	High	24.88 ± 0.84 a	24.09 ± 0.43 a	22.23 ± 0.62 b
		Low	25.99 ± 1.47 a	22.06 ± 1.23 b	22.16 ± 0.34 b
Panicle length (cm)		High	17.36 ± 3.16 a	17.83 ± 2.01 a	15.42 ± 3.11 b
		Low	16.53 ± 2.27 a	16.42 ± 1.35 a	16.35 ± 0.81 a
Spikelet density/10 cm		High	83.29 ± 7.57 b	87.14 ± 8.64 b	90.64 ± 8.71 a
		Low	80.61 ± 6.52 b	87.88 ± 7.65 a	82.45 ± 6.55 b
Panicle type index		High	0.47 ± 0.01 a	0.45 ± 0.01 b	0.43 ± 0.01 c
		Low	0.48 ± 0.01 a	0.46 ± 0.01 b	0.44 ± 0.01 c

Values marked by the same letter do not differ according to Tukey’s honestly significant difference at a 5% confidence level.

**Table 8:** Basic information of the test materials.

Cultivars	Growth Days (d)	Plant Height (cm)	Tiller Ability	Panicle Type	Breeding Institution
Beijing 1	154	105.0	Intermediate	Erect	Shenyang Agricultural University, Shenyang, Liaoning, China
Beijing 2	155	106.4	Strong	Erect	Shenyang Agricultural University, Shenyang, Liaoning, China
Beijing 3	154	101.4	Strong	Erect	Shenyang Agricultural University, Shenyang, Liaoning, China
Beijing 1501	159	104.9	Moderate	Semi-erect	Shenyang Agricultural University, Shenyang, Liaoning, China
Beijing 1604	152	103.6	Strong	Erect	Shenyang Agricultural University, Shenyang, Liaoning, China
Shendao 47	155	105.0	Strong	Semi-erect	Shenyang Agricultural University, Shenyang, Liaoning, China
Liaojing 1415	155	104.6	Moderate	Semi-erect	Liaoning Rice Research Institute, Shenyang Agricultural University, Shenyang, Liaoning, China
Liaojing 401	158	105.2	Intermediate	Erect	Liaoning Rice Research Institute, Shenyang Agricultural University, Shenyang, Liaoning, China
Liaojing 399	154	110.1	Moderate	Erect	Liaoning Rice Research Institute, Shenyang Agricultural University, Shenyang, Liaoning, China
Liaojing 212	158	110.1	Intermediate	Erect	Liaoning Rice Research Institute, Shenyang Agricultural University, Shenyang, Liaoning, China
Tiejing 7	156	91.1	Moderate	Erect	Tieling Academy of Agricultural Sciences, Tieling, Liaoning, China
Tiejing 11	159	100.9	Strong	Semi-erect	Tieling Academy of Agricultural Sciences, Tieling, Liaoning, China
Tiejing 20	155	103.4	Strong	Semi-erect	Tieling Academy of Agricultural Sciences, Tieling, Liaoning, China
Jindao 108	156	105.4	Strong	Erect	Panjin North Agricultural Technology Development Co., Ltd., Panjin, Liaoning, China
Jindao 201	157	92.9	Strong	Erect	Panjin North Agricultural Technology Development Co., Ltd., Panjin, Liaoning, China
Jindao 104	159	94.5	Strong	Erect	Panjin North Agricultural Technology Development Co., Ltd., Panjin, Liaoning, China
Yanjing 765	156	100.1	Strong	Semi-erect	Liaoning Institute of Saline-alkali Land Utilization, Panjin, Liaoning, China
Yanjing 662	157	97.6	Strong	Erect	Liaoning Institute of Saline-alkali Land Utilization, Panjin, Liaoning, China
Fuhe 5	156	100.0	Moderate	Semi-erect	Liaoning Dong-A Seed Industry Co., Ltd., Shenyang, Liaoning, China
Meifengdao 669	154	108.8	Moderate	Semi-erect	Liaoning Dong-A Seed Industry Co., Ltd., Shenyang, Liaoning, China

List of the tested varieties.

## Data Availability

The data used to support the findings of this study can be made available by the corresponding author upon request. The data are not publicly available due to the fund information and other papers have not been published, this data will not be uploaded.
